# Adaptive rewiring evolves brain-like structure in weighted networks

**DOI:** 10.1038/s41598-020-62204-7

**Published:** 2020-04-08

**Authors:** Ilias Rentzeperis, Cees van Leeuwen

**Affiliations:** 1 KU Leuven, Leuven, Belgium; 2 University of Technology Kaiserslautern, Kaiserslautern, Germany

**Keywords:** Dynamical systems, Network models

## Abstract

Activity-dependent plasticity refers to a range of mechanisms for adaptively reshaping neuronal connections. We model their common principle in terms of adaptive rewiring of network connectivity, while representing neural activity by diffusion on the network: Where diffusion is intensive, shortcut connections are established, while underused connections are pruned. In binary networks, this process is known to steer initially random networks robustly to high levels of structural complexity, reflecting the global characteristics of brain anatomy: modular or centralized small world topologies. We investigate whether this result extends to more realistic, weighted networks. Both normally- and lognormally-distributed weighted networks evolve either modular or centralized topologies. Which of these prevails depends on a single control parameter, representing global homeostatic or normalizing regulation mechanisms. Intermediate control parameter values exhibit the greatest levels of network complexity, incorporating both modular and centralized tendencies. The simulation results allow us to propose diffusion based adaptive rewiring as a parsimonious model for activity-dependent reshaping of brain connectivity structure.

## Introduction

From neuronal synapses to white matter tracts^1^, brain anatomical networks are characterized by the structural properties of small-worldness^2,3^, modularity^4,5^ and rich club organization^6,7^. The pervasiveness of these properties raises the question whether they result from a common principle^5,8^. We proposed that these properties are the product of adaptive rewiring^9–13^^, for a review^^14^. Adaptive rewiring captures a crucial property of how the brain’s anatomical network is shaped over time. Whereas the mechanisms that shape the brain network show great variety, as they encompass brain growth^15^, development, as well as learning^16^^,for a review^, they are alike in their common dependency on the network’s *functional connectivity*, i.e. the statistical dependencies between the nodes’ activities^12,17^. Adaptive rewiring formalizes this dependency in terms of graph theory, as it encompasses adding shortcut links to network regions with intense functional connectivity while pruning underused ones. These dynamical rewirings could be regarded as adaptive network optimization to function^18^.

Whereas adaptive rewiring represents the dependency of structural connectivity on network activity, the reverse, viz. the dependency of activity on structural connectivity, has been the focus of intense investigation. Recently, it was proposed that a simple, linear equation can predict brain activity from anatomical connectivity^19^. The proposal describes traffic on a fixed anatomical network in terms of random walks. This allows the amount of traffic to be stochastically approximated in terms of diffusion on the graph. Jarman and colleagues^11^ adopted a similar principle in an adaptive rewiring model. They represented the amount of flow transferred between nodes by a heat kernel. During each rewiring step, an edge with low flow is pruned while an unconnected pair of nodes with high flow between them is connected.

The rewiring algorithm is tuned by two parameters: random rewiring (p_random_) and rewiring rate (τ). The parameter p_random_ is the proportion of rewirings performed at random, rather than according to the adaptive rewiring criterion. This represents the proportion of rewirings made in adaptation to neural noise. The parameter τ is the elapsed time in the diffusion process before a rewiring is made. We speak of fast rewiring when τ is small, and slow rewiring when τ is large. The parameter may represent global homeostatic or normalizing regulation mechanisms^for a review^ ^20^.

An important shortcoming of the adaptive rewiring model as proposed by Jarman and colleagues^11^ is that it uses binary networks to represents brain anatomy. Notwithstanding the popularity of binary graphs in brain network analysis, networks with differentially weighted connections offer a more appropriate representation of the sophisticated data on brain connectivity from contemporary tract-tracing and other imaging studies^2^. Recent measures are applied on more realistic, differentially weighted anatomical connections; thus a corresponding graph representation of the brain is necessary. Robust convergence to small world topologies is not automatically guaranteed for adaptive rewiring of differentially weighted networks; rewiring of low-weight connections may not impact the network flow, whereas highly weighted ones may resist pruning^21^.

Here, we first probe whether graph diffusion can drive the evolution of brain-like connectivity structures in weighted networks. Presynaptic weights on single neurons have traditionally been modeled as normally distributed, but recent studies favor lognormal distributions, exhibiting a long tail of strong connections^22,23^. We therefore compare rewiring in normal and lognormal weight distributions.

Our results show that adaptive rewiring according to network diffusion establishes ‘brain-like’ network structure for both normal and lognormal weight distributions. More specifically, adaptive rewiring steers random networks to intricately structured, small-world patterns for a wide range of rewiring rates, even with large proportions of random rewirings. The rewiring rate dictates the spread of diffusion from each node, with fast rewiring affecting mostly neighbors and slow rewiring having a more widespread effect. The speed of rewiring naturally determines the connectivity pattern of the network after rewiring. Fast rewiring biases rewiring towards local structures and produces modular connectivity patterns, whereas slow rewiring biases the network evolution towards global structures, resulting in centralized connectivity patterns. In the intermediate range of rewiring rates, there is a transition zone, in which networks show the greatest variability. Nodes with higher degrees show a preferential attachment to each other, exhibiting the so-called rich club effect. However, larger weights are not preferentially attached to this subnetwork. This pattern of attachment is another connectivity feature that is shown to exist in the brain^24,25^. Overall, due to its robust convergence to brain-like topologies and its small number of tunable parameters, graph diffusion is a parsimonious model linking anatomy with functional connectivity.

## Materials and Methods

### Graph preliminaries

A graph is defined as an ordered triple *G* = (*V*, *E*, *W*), where *V* denotes the set of nodes (or vertices), *E* the edges (or connections) between them, and *W* the set of edge weights, $W=\{{w}^{ij}\in {{\mathbb{R}}}_{\ge 0}|(i,j)\in E\}$, i.e. only nonnegative weights are used. The cardinalities, *|V|* = *n* and *|E|* = *m*, express the total number of nodes and connections respectively. The connectivity pattern of *G* can be conveniently represented by an *n*X*n* adjacency matrix, *A*, with its entries denoting the weights between nodes, i.e. *A*_*ij*_ = *w*^*ij*^. *w*^*ij*^ = 0 signals that edges *i* and *j* are not adjacent. If *w*^*ij*^ > 0 then nodes *i* and *j* are adjacent; the greater the value of the weight the stronger the connection between nodes. In the case of a binary network, the weights and the entries of the adjacency matrix can take only two values, 0 or 1; *A*_*ij*_ = 1 indicates that nodes *i* and *j* are adjacent ((*i*, *j*) ∈ *E*), and *A*_*ij*_ = 0 that they are not ((*i*, *j*) ∉ *E*). In this paper both binary and weighted graphs are undirected and simple (there are no self loops), meaning *A* is symmetric (*A*_*ij*_ = *A*_*ji*_) and zero in its diagonal entries (*A*_*kk*_ = 0) respectively. The strength of a node *j* is the sum of the weights from the edges incident to it, and is obtained by summing the rows or the columns of the adjacency matrix: ${s}_{j}={\sum }_{i=1}^{n}{A}_{ij}$. In the case of binary networks, the summation indicates the degree *d*_*j*_ of node *j*.

### The graph laplacian matrix

The graph Laplacian is defined as *L* = *D* − *A*, where *D* is a diagonal matrix having the strengths (or degrees for binary adjacency matrices) of the nodes in its diagonal entries (*D*_*ii*_ = *s*_*i*_). It arises naturally in optimization problems such as graph partitioning^26^ or nonlinear dimensionality reduction^27^. It also emerges as the discrete analogue of the Laplace-Beltrami operator (∇^2^*f*) in the heat flow equation for graphs. The normalized graph Laplacian is defined as ${\mathcal L} ={D}^{-1/2}L{D}^{-1/2}$, with ${D}_{ii}^{-1/2}=0$ for *s*_*i*_ = 0. Its entries take the values:

1 $${{\mathcal{L}}}_{ij}=\{\begin{array}{c}1\,if\,i=j\\ \frac{-{A}_{ij}}{\sqrt{{s}_{i}{s}_{j}}}\,if\,(i,j)\in E\\ 0\,otherwise\end{array}$$

We use the normalized graph Laplacian, a more appropriate operator for graphs that are not regular, that is graphs with nodes that do not necessarily have the same strength (or degree). This is because the eigenvector of the normalized graph Laplacian corresponding to the zero eigenvalue captures the graph irregularity: ${\lambda }_{0}=0,\,{v}_{0}={[\sqrt{{s}_{1}},\ldots ,\sqrt{{s}_{n}}]}^{T}$^28^. Thus, throughout the paper, any mention of the Laplacian refers to the normalized version.

The Laplacian is a symmetric positive semidefinite matrix with real values: ${ {\mathcal L} }_{ij}={ {\mathcal L} }_{ji}$ and ${z}^{T} {\mathcal L} z\ge 0,\,\forall z\in {{\mathbb{R}}}^{n}$; consequently, its eigenvalues are nonnegative and real and its eigenvectors form an orthonormal set. The spectral decomposition of the Laplacian matrix is ${\mathcal L} =V\Lambda {V}^{T}$, where $\Lambda =diag\,({\lambda }_{0},{\lambda }_{1}\ldots {\lambda }_{n})$ is a diagonal matrix, having in its diagonal entries the eigenvalues of ${\mathcal L}$ ($0={\lambda }_{0}\le {\lambda }_{1}\le \ldots \le {\lambda }_{n}\le 2$) and $V=[{v}_{0}{v}_{1}\ldots {v}_{n}]$ is an *n*X*n* matrix having as its columns the corresponding orthonormal eigenvectors (${\mathcal L} {v}_{i}={\lambda }_{i}{v}_{i}$).

The eigenvalues and eigenvectors of the Laplacian yield valuable information about the graph they are derived from^29^. The multiplicity of zero indicates the number of connected components in the graph; a single zero eigenvalue corresponds to a connected graph, two zero eigenvalues to a disconnected graph with two components, and so on. The second smallest eigenvalue, called the Fiedler value, and the largest one are related to several graph properties such as the graph’s connectivity, diameter and convergence to a stationary probability distribution for random walks^30–33^. The values of the Fiedler vector, the eigenvector corresponding to the smallest nonzero eigenvalue, can be used to sort nodes so that the ones close to each other belong to the same community, and accordingly partition a graph^26,34^. Nodes are sorted in the same order as the elements of the Fiedler vector in figures showing adjacency matrices.

### Heat kernel

The heat equation is a partial differential equation that describes how heat (or another quantity) is spatially dissipated over time in a medium. It is defined as:

2 $$\frac{\partial h(t,\,{x}_{1,\ldots ,}\,{x}_{n})}{\partial t}=a{\nabla }^{2}h(t,\,{x}_{1,\ldots ,}\,{x}_{n})$$

where *h* is a function of n spatial variables *x*_1_,*…*,*x*_*n*_ and time variable *t*. *α* is a positive constant, representing the diffusivity of the medium; the greater the value of *α* the faster the diffusion. We set it to 1. ∇^2^ is the Laplacian operator, also called the divergence of the gradient.

The heat equation based on the graph Laplacian matrix is similarly describing the variation in the flow of heat (or energy or information) within a graph over time:

3 $$\frac{\partial h(t)}{\partial t}=- {\mathcal L} h(t)$$

The heat kernel, *h*(*t*), and the graph Laplacian, ${\mathcal L}$, are both *n*X*n* matrices describing the flow of heat across edges and its rate of change respectively. *h*(*τ*)_*ij*_ is the amount of heat transferred from node *i* to node *j* after time *t* = τ. The heat equation has an explicit solution:

4 $$h(t)={e}^{-t {\mathcal L} }$$

As was shown in the previous section, ${\mathcal L}$ can be decomposed into its eigen-spectrum. The heat kernel can then be written as:

5 $$h(t)=V{e}^{-t\Lambda }{V}^{T}=\mathop{\sum }\limits_{i=0}^{n-1}{e}^{-{\lambda }_{i}t}{v}_{i}{v}_{i}^{T}$$

The dynamics for which diffusion spreads in the graph are based on the eigenvalue/eigenvector pairs of the Laplacian matrix. In the summation the contribution of each pair is not the same, but depends on the magnitude of the eigenvalue. For a connected graph, when *t* is close to zero then $h(t)\approx I- {\mathcal L} t$. In this case, diffusion is dominated by local connections. In contrast, for large t values, the heat kernel can be approximated by the contribution of the eigenvalue/eigenvector pairs with the smallest eigenvalues. For large t, the global structure is favored^35^. S1 Appendix gives some more intuition on the heat kernel. During an adaptive rewiring process -we discuss subsequently- *t* has a fixed value (*t* = τ); we refer to it as the rewiring rate (τ). τ is used at each nonrandom iteration and signifies the time elapsed during the diffusion process before a decision is made on which edge to add and which to remove.

### Adaptive rewiring algorithm

With the adaptive rewiring algorithm employed in this paper, we seek to probe the effects that a simple self-organizing rule will have on the properties of an initially randomly connected network. The algorithm below is the same as the one in Jarman and colleagues^11^, extended to both binary and weighted networks. An equivalent definition that is in direct correspondence with the code implementation is also described in S2 Appendix.

We formulate the algorithm as a rewiring process embedded within a dynamical system evolving over time. We start with a random Erdös–Rényi network with *|V|* = *n* nodes. At each rewiring iteration we select, with uniform random probability, a node, *k* from the set of nodes in the graph that are of nonzero degree but also not connected to all other nodes $(k\in V|0 < {d}_{k} < n-1)$. Then we select node *j*_1_ from the set of nodes that are not connected to *k*, $({j}_{1}\in \{j\in V|(j,k)\notin E\})$, and node *j*_2_ from the set of nodes that are connected to *k*, $({j}_{2}\in \{j\in V|(j,k)\in E\})$. We delete the edge, (*k*, *j*_2_) and add the edge (*k*, *j*_1_). In the case of weighted networks we use the weight of the previously connected edge (*k*, *j*_2_) for the edge (*k*, *j*_1_). The selection of nodes *j*_1_ and *j*_2_ is different for the random and heat diffusion based rewirings. In the case of random rewirings the selection of both *j*_1_ and *j*_2_ is random and uniform among the elements of each set described above. In the case of heat diffusion rewiring we calculate the heat kernel, *h*(*τ*), of the adjacency matrix. *j*_1_ is selected such that from all the nodes not connected to *k*, it is the one with the highest heat transfer with *k*. *j*_2_ is selected such that from all the nodes connected to *k*, it is the one with the lowest heat transfer with *k*.

The whole iteration of rewirings is defined as follows: Heat diffusion based rewiring takes place at times t_1_ = τ, t_2_ = 2τ,…, t_Ν_ = *M*τ, with *M* being the total number of this kind of rewirings. In addition, random rewirings are interspersed in between the heat diffusion rewirings. Whereas heat diffusion-based rewirings need evidence for a rewiring decision to accumulate over duration τ, this is not the case for random rewirings. Thus we assume random rewirings are approximately instantaneous compared to the heat based rewirings and do not assign them any time duration. At time t = 0 the heat kernel is initialized to the identity matrix: *h*(0) = *I*. With probability p_random_ the rewiring is random; with probability 1- p_random_ the rewiring is based on heat diffusion in the network; for both cases the criteria are explained above. At the onset of the n^th^ instantiation of the heat diffusion rewiring, the heat kernel is reset to *h*(0) = *I* and then heat diffusion evolves in the network for a duration of t_n_ − t_n-1_ = τ; a rewiring is made based on the state of *h*(*τ*). We repeat until we reach the total number of preset rewirings.

The rewiring rate, τ, is controlling how long the diffusion process lasts until a decision is made and is constant throughout the rewiring process. Small τ values affect mostly node pairs with direct connections since the diffusion process does not have enough time to integrate along the network before a rewiring is made. However for longer τ values, node pairs that are not directly connected also gain significance since diffusion spreads more globally.

Simulations and analyses were performed on 100-node networks (average degree was 18.24). Normally distributed weights were sampled from the probability density function of the normal distribution:

6 $$p(x)=\frac{1}{\sigma \sqrt{2\pi }}{e}^{-\frac{{({\rm{x}}-\mu )}^{2}}{2{\sigma }^{2}}}$$

with *μ* = 1 and *σ* = 0.25. Negative values sampled from this density function were rare, but when encountered were set to zero. Lognormally distributed weights were sampled from the probability density function of the lognormal distribution:

7 $$p(x)=\frac{1}{\sigma \sqrt{2\pi }}{e}^{-\frac{{(\mathrm{ln}(x)-\mu )}^{2}}{2{\sigma }^{2}}}$$

with *μ* = 0 and *σ* = 1. For both distributions the weights were divided by the maximum weight of the network so that the values are between 0 and 1. Different parameters and normalizations were tested without any significant differences in the results.

**Figure 1:**
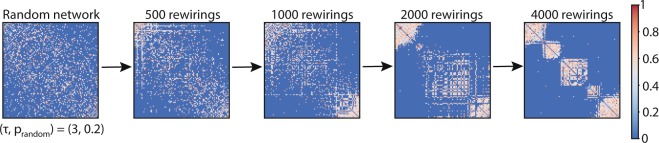
Example of an initially random network at progressive stages of rewiring.

Unless otherwise stated the number of nodes, n, in our analysis was 100 and the number of connections was *m* = ⌊*2* *log*(*n*)(*n* − *1*)⌋ = 912, which is twice the critical density of a random Erdös–Rényi graph for which it is connected with probability 1^36^. Most simulations vary two parameters from the rewiring algorithm: p_random_ and τ. For each combination of parameter values, we run the rewiring algorithm 100 times. Each run involves 4000 rewirings, since by then networks typically have diverged from a random connectivity pattern. We also examined the rewiring algorithm’s behavior for approximately asymptotic τ values; for the sake of convenience we denote them ε = 10^−15^ and δ = 10^15^. Unless otherwise stated, the figures show mean values of these 100 runs. Figure 1 shows an example evolution of a network at progressive stages of rewiring.

The network before rewiring is randomly connected. The weights of the connections are normally distributed. The rewiring parameters throughout ((τ, p_random_) = (3, 0.2)) give rise to a modular network. The networks are shown in the form of adjacency matrices; the color scaling indicates the weights of the connections.

Description of the different metrics (clustering coefficient, efficiency, small-worldness, modularity, assortativity coefficient and rich club coefficient for binary and weighted networks) is found in S3 Appendix. Unless otherwise stated, the analyses were programmed in Python. The code package of the functions producing the rewired adjacency matrices, as well as the metrics described in Supplementary Materials are publicly available in a GitHub repository (https://github.com/rentzi/netRewireAnalyze). The interested reader can run the whole gamut of simulations in the jupyter notebooks included in the toolbox package.

## Results

We divide this section into different subsections according to the metrics provided. First, we show that, similarly to binary networks, both normally and lognormally weighted networks evolve to have small world structures for a wide range of p_random_ and τ values. Networks with identical small world values can have diverging topological structures depending on the value of τ. The modularity index and a degree outlier measure differentiate between these topologies. Using these metrics we show that, typically for small τ values, all types of networks evolve to have densely clustered, modular structure, while for larger τ values they have a centralized connectivity pattern, where only a small subset of the nodes acts as the network backbone. For a narrow range of τ values in between the ones producing modular and centralized connectivity patterns, rewired networks show great variability in their connectivity structures, ranging from modular to centralized. Finally, networks in the modular and in-between states have distinct topological and weighted rich club behavior similarly to what is observed in empirical studies of the human brain.

### Small-worldness

**Figure 2:**
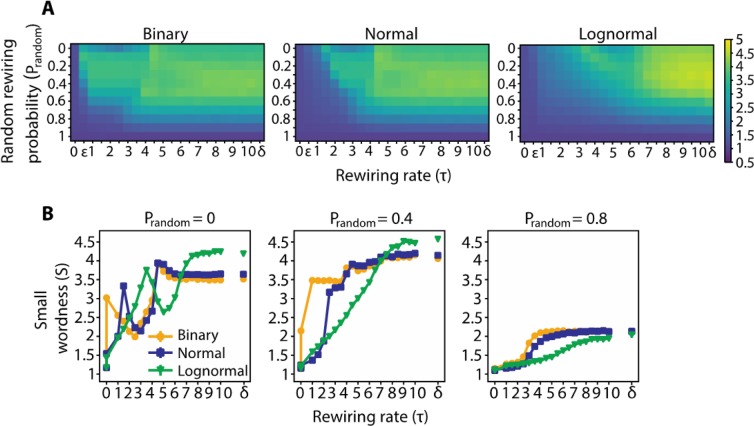
Networks with binary, normal and lognormal weight distributions develop into small worlds. (**A**) From left to right, small world values (*S*) for networks with binary, normal and lognormal weight distributions for different rewiring rates (τ) and random rewiring probabilities (p_random_). ε = 10^−15^, δ = 10^15^. (**B**) *S* as a function of τ for different values of p_random._ The s.e.m lines are not visible since they do not exceed the markers.

Small-worldness (*S*) is observed for all networks following rewiring, albeit with non-identical profiles across the system parameters τ and p_random_ (Fig. 2A). *S* increases more steeply as a function of τ for binary networks than for normal and lognormal ones, the latter showing the most gradual increase (Fig. 2B). For τ = τ_plateau_ (different for each of the three weighting regimes), *S* reaches a maximum value, *S*_*max*_, henceforth sustaining it. *S*_*max*_ decreases significantly for p_random_ > 0.4. (Fig. 2B) since the increased random rewiring partially cancels out the clustering created by diffusion.

Small worldness is traditionally defined as the ratio of the network’s clustering coefficient (*C*) over its path length (*L*). For our purposes, we use efficiency (*E*) instead of *L*. *E* which is the inverse of *L*, indicates the ease of flow of information between edges and is a more natural metric for weighted connections: the larger the weight of an edge the greater the flow and the more efficient the paths that include that edge. In this context *S* is equal to the product of *E* and *C* (Eq. 8 in S3 Appendix). An increase in a network’s clustering coefficient or efficiency induces by definition an increase in its small worldness. We probed the dynamics of *C* and *E* that drove up the small worldness of the networks. We found that along τ, *S* shows a strong positive correlation with *C* (ρ_binary_ = 0.91, ρ_normal_ = 0.96, ρ_lognormal_ = 0.97; ρ = Pearson coefficient) and a moderately negative one with *E* (ρ_binary_ = −0.42, ρ_normal_ = −0.52, ρ_lognormal_ = −0.31). In the Watts and Strogatz model^37^, the authors start with a structured network and parametrically swap the connections constituting the structure (the ones where neighboring nodes are connected) with random ones. This induces a significant decrease in *L* but a more moderate one in *C*, resulting in an increase in the small worldness of the network following rewiring. Our model uses the reverse process, in which rewiring induces a random network configuration to morph into a more structured one, and thus becoming small world due to an increase in *C* (S4 Appendix describes in more detail the dynamics of *C* and *E*).

### Modularity

**Figure 3:**
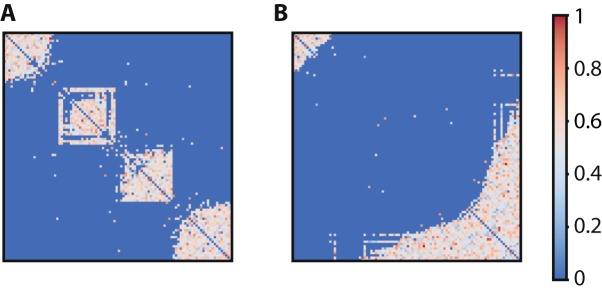
Networks can have the same small world value but diverging connectivity pattern. (**A**) Example of a modular network (τ = 3) where nodes within clusters are densely intraconnected but sparsely interconnected between clusters. (**B**) Example of a centralized network (τ = 5) where there is a degree imbalance between nodes, with the vast majority of the nodes having very few connections and the rest being heavily connected, acting as hubs. Both networks have the same small world value (*S* = 3.4) despite their different topological characteristics. In both cases p_random_ is 0.2 and the weight distribution is normal. The color scaling indicates the weights of the connections.

Small worldness characterizes networks with widely diverging topological qualities^38^. For example, both of the adjacency matrices in Fig. 3 have the same *S* value. However, the one in Fig. 3A consists of clusters with dense intragroup and sparse intergroup connections. This characterizes modular connectivity patterns. On the other hand, the one in Fig. 3B comprises of a small number of nodes that are heavily connected, acting as hubs with the rest having very few connections. This is a feature of centralized topologies. Newman’s^39^ modularity index (*Q*) differentiates between the two networks of Fig. 3 (*Q*: 0.70 vs. 0.22 for Fig. 3A,B respectively).

**Figure 4:**
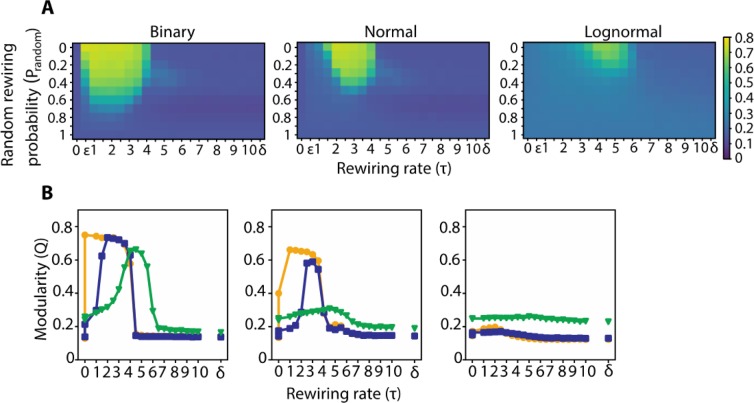
Modularity profiles between networks with binary, normal and lognormal weight distributions have similar shape. (**A**) From left to right, Modularity index *Q* for networks with binary, normal and lognormal weight distributions for different values of the system parameters τ and p_random_. (**B**) *Q* as a function of τ for different p_random_. The s.e.m. lines do not exceed the markers.

To identify the clusters or modules, we use the spectral algorithm introduced by Newman^39^ (S3 Appendix). In the case of the weighted networks we used the strengths instead of the degrees of the modularity matrix the algorithm aims to optimize (Eq. S9). Binary, normally and lognormally weighted networks develop modularity as a function of τ and p_random_ in a similar fashion (Fig. 4A). Generally, *Q* initially increases as a function of τ, reaching a plateau, but eventually drops off to a value close to that of a random network (τ = 0) (Fig. 4B). Binary networks reach their maximum *Q* faster (τ = 10^−15^) compared to normal (τ = 2), and lognormal networks (τ = 4) (Fig. 4B). This is directly related to the behavior of *h*(*τ*) for different weighted regimes (S1 Appendix). In the case of binary networks, heat from each node spreads to its neighbors the fastest (smallest τ value). This effects in the creation of clusters after a number of iterations and subsequently in networks with high *Q* values. For normal and lognormal networks the τ values that are critical for *h*(*τ*) to induce clusterings are greater. For larger τ values (slower rewiring rate) heat is diffused throughtout the nodes almost homogeneously, effectively breaking down modularity (example in Fig. S1, *h*(*τ* = 10)).

Furthermore, the binary networks sustain the maximum *Q* value for a greater range of τ values compared to the two weighted regimes, with the lognormal networks having the smallest range. For all networks, random rewiring diminishes the maximum *Q* value, with the binary networks being the most resistant and the lognormal ones the most exposed to this effect (Fig. 4B). The pattern of results presented here were identical with the ones from a multilevel algorithm^40^ implemented by the igraph toolbox^41^ (compare Fig. S4 with Fig. 4A).

### Degree and strength distributions of modular and centralized networks

**Figure 5:**
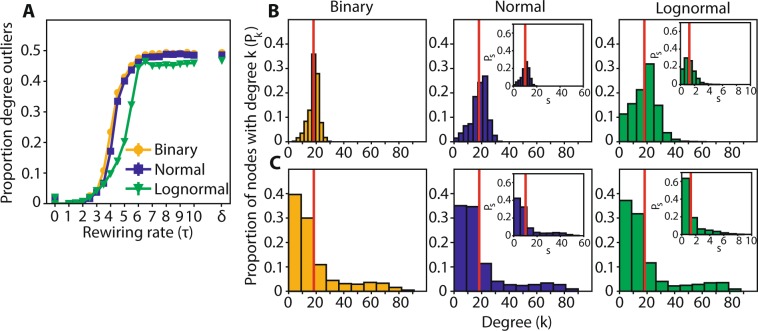
Degree distribution for modular networks is close to the mean value; for centralized networks it is heavy tailed. (**A**) Proportion of nodes with outlier degrees as a function of τ for binary, normal and lognormal networks. The s.e.m. lines do not exceed the markers. (**B**) From left to right, the distribution of degrees for binary, normal and lognormal networks (τ_binary_ = 2, τ_normal_ = 3, τ_lognormal_ = 4.5) which are modular. Inset plots show the strength distributions. C. Same as in B, but for centralized networks (τ_binary_ = 5, τ_normal_ = 5, τ_lognormal_ = 7). In all cases p_random_ = 0.2.

For a fixed number of total connections, centralized networks can be distinguished from modular ones according to the number of nodes with degrees deviating significantly from the mean. Centralized networks have a small number of high-degree nodes acting as hubs and, correspondingly, a large number of nodes with low degree. Both of these groups constitute outliers in the connectivity distribution. Hence, the proportion of outliers characterizes the centralization in the network. Degrees in the vicinity of the mean were established as <k> ± 3σ_κ_, where <k> is the mean degree of the network and σ_κ_ its dispersion. We used the Poisson distribution with mean <k> to calculate the value of the dispersion parameter (σ_κ_ = <k>^1/2^). A Poisson distribution is a suitable baseline distribution since random networks by virtue of their construction have most node degrees close to the mean and are well approximated by it. We probed how the proportion of outliers changes with τ, and which range of τ gives rise to centralized networks. We found that the proportion of outliers in networks as a function of τ follows a sigmoid function, with binary and normal networks having almost identical values but lognormal ones having a more gradual transition (Fig. 5A).

Both the modularity and outlier analyses are in accord with each other: the range of τ values for which the networks have a small number of outliers includes the τ values for which the *Q* value is at its highest (it also includes τ values close to zero for which the *Q* value is small where the network is in a state starting to deviate from randomness but not quite reaching a definite structure). For τ values greater than this modular range, networks qualify as centralized according to our outliers criterion. Modular and centralized networks have different degree distributions, the former’s being concentrated around the mean (Fig. 5B) and the latter’s having a large spread with a heavy tail (Fig. 5C). The τ values giving rise to the distributions in Fig. 5B,C are typical for modular and centralized topologies respectively. Furthermore, weighted networks show the same degree and strength distributions (Fig. 5B,C, inset plots).

We probed the heavy tail degree and strength distributions of representative centralized networks (τ_binary_ = 5, τ_normal_ = 5, τ_lognormal_ = 7, in all cases p_random_ = 0.2). The power-law and lognormal distributions were a better fit than the exponential one (Fig. S3). Power law distribution functions are of the form *P*(*k*) ~ *k*^*−α*^. For the degree distributions the exponent *α* that fitted best the data was 1.7, for the strength distributions it varied between 2 and 2.6 for the different types of networks (S5 Appendix has a detailed description of the analysis). Note that this analysis shows that power law is a better fit than the exponential distribution, not necessarily that diffusion rewiring can generate scale-free networks. We used publicly available code for this analysis^42^.

### Assortativity and rich club structure

We probed the possibility that the rewiring process favors homophily by measuring the topological assortativity coefficient, *r*. Networks with positive *r* are assortative, meaning that nodes with similar degrees tend to connect. Networks with negative *r* are disassortative. In this case nodes with dissimilar degrees are more prone to connect compared to a randomly connected network with the same degree distribution. We found that for modular networks, *r* shows weak or zero assortativity; centralized networks dip into the disassortative realm (Fig. S5).

We further measured the rich club metrics of the networks. Topological rich club, *Φ*(*k*), refers to the tendency of typically high degree nodes to connect with each other; when its normalized counterpart, *Φ*_*norm*_(*k*), is above the baseline (greater than 1), then the subset of nodes with degree greater than *k* is more densely interconnected compared to *Φ*_*random*_(*k*), a random control with the same degree distribution. The rich club coefficient is not trivially connected to assortativity, since a disassortative network could still be rich club and vice versa^43^. We tested the rich club metrics for τ values that produce modular and centralized connectivity patterns. We also tested the rich club behavior of networks produced from τ values in the middle of the phase transition from modular to centralized networks (τ_transition_). This τ_transition_ point is the one that gives the largest derivative value on each of the sigmoid curves in Fig. 5A (inflection point). In all cases, τ_transition_ gives rise to networks with the highest variability of network topologies compared to all the other τ values, varying from modular to centralized ones.

**Figure 6:**
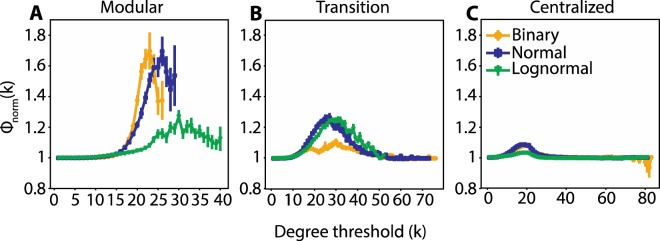
Weighted networks show the most prominent topological rich club for rewiring rates in the modular and transition ranges, binary networks only in the modular range. Topological normalized rich club, *Φ*_*norm*_(*k*), for binary, normal and lognormal networks in the (**A**) modular (τ_binary_ = 2, τ_normal_ = 3, τ_lognormal_ = 4.5) (**B**) transition (τ_binary_ = 4.1, τ_normal_ = 4.15, τ_lognormal_ = 5.5) and (**C**) centralized (τ_binary_ = 5, τ_normal_ = 5, τ_lognormal_ = 7) state. In all cases p_random_ = 0.2. Vertical lines indicate s.e.m.

By definition, the number of nodes in a rich club as a function of degree threshold is monotonically decreasing. We found that the decrease profiles are similar for binary and weighted networks, but varied in their rate across different τ values with modular networks showing the steepest rate of decrease, centralized the most gradual one and transition an intermediate one (Fig. S6). Typically, for all τ values tested, *Φ*(*k*) is greater than *Φ*_*random*_(*k*) for a range of intermediate degree thresholds, with transition networks showing the widest range of degree thresholds for which this difference is significant and centralized networks the narrowest one (Fig. S7). The greatest divergence between *Φ*(*k*) and *Φ*_*random*_(*k*) indicated by their ratio (*Φ*_*norm*_(*k*)) is shown for binary modular networks and for both modular and transition weighted networks (Fig. 6).

**Figure 7:**
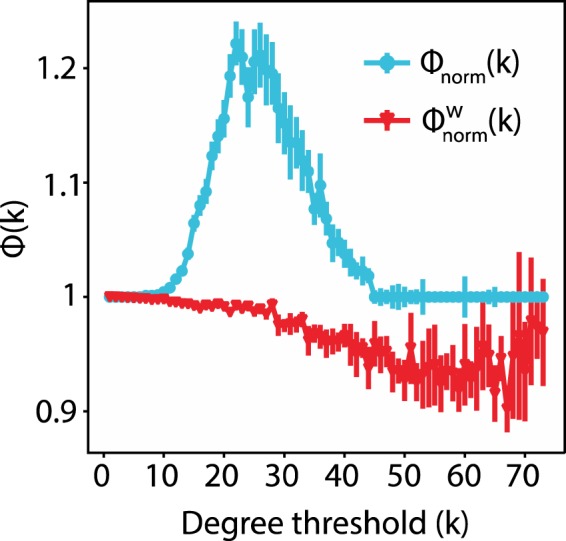
Topological and weighted rich club coefficients diverge similarly to physiological data. Topological and weighted rich club coefficient for the normal network at τ_transition_ = 4.15, p_random_ = 0.2. Vertical lines indicate s.e.m.

For normal and lognormal networks, we evaluated the normalized weighted rich club coefficient, *Φ*_*w*,*norm*_(*k*). *Φ*_*w*,*norm*_(*k*) above the baseline indicates that the edges of the rich club nodes have larger weights compared to a random control network (a network with the same topology but randomly reshuffled weights). We found that for the lognormal networks *Φ*_*w*,*norm*_(*k*) did not deviate from 1; for the normal networks, *Φ*_*w*,*norm*_(*k*) similarly hovered around 1 in the modular and centralized regimes. Hence for these states the rewiring process does not distribute the larger weights preferentially to the high degree nodes. In general the data show that there is a distinction between the topological and weighted measures; the former being above baseline for a range of degrees, but not the latter. For normally distributed networks, this distinction becomes more pronounced in the transition zone, where *Φ*_*w*,*norm*_(*k*) tilts below baseline for nodes with larger degrees (*k* > 20) (Fig. 7).

Taken together, these topological and weighted coefficient profiles are in agreement with anatomical data on the human brain. Specifically, van den Heuvel and colleagues^25^ mapped human brain structural connectivity by estimating via diffusion tensor imaging (DTI) the number of connecting white matter streamlines between 1,170 subdivisions of the cortex. In a subsequent analysis of the same data, based on topology the human network exhibits rich club behavior, but its weighted counterpart does not (Fig. S8^; reproduced from^ ^24^), exhibiting the same qualitative properties as the normal –and the lognormal to a lesser extent- network at τ_transition_ (compare Fig. 7 to Fig. S8).

## Discussion

### Graph diffusion and other rewiring models

Adaptive rewiring robustly drives random binary networks to complex architectures matching brain anatomy on the key characteristics of small world structure, modular and centralized topology. A test of generality for adaptive rewiring is whether it can drive networks to brain-like architectures with differentially weighted connections. Our first observation is that the adaptive rewiring model passes this test.

In previous adaptive rewiring models, functional connectivity was represented as synchronous activity^9,10,12,13,21,44,45^ of linearly coupled nonlinear oscillators^46^ or, somewhat more realistically, by spiking model neuron synchrony^47,48^. While these representations more closely resemble artificial neural networks than the current graph diffusion model, their shortcomings are twofold. They rely on arbitrary functional connectivity dynamics, whereas a representation in terms of diffusion is empirically adequate^19^. Moreover, rewiring in the older models involved a global search for the best (i.e. most synchronized) rewiring target. Even though this problem was remedied in Jarman and colleagues^44^ by taking rewiring distance into account, this introduced arbitrary assumptions about the spatial embedding of these networks. Jarman and colleagues^11^ developed the graph-based solution to this problem adopted here, based on the speed of rewiring relative to that of the heat diffusion (τ). The lower τ is, the faster the rewiring, and hence the narrower the network search for the best rewiring candidate; higher τ values offer slower rewiring rates, allowing the search to be incrementally broader. The possibility to choose an appropriate value for τ offers a natural control parameter to determine whether the resulting network structure tends to be modular or centralized. In biological systems, this choice may reflect global homeostatic forms of plasticity regulating the network’s dynamics^for a review^ ^20^.

The current graph diffusion model offers an explanation on the topological structure of the brain just with a single parameter, τ (since for a wide range of p_random_ we get the same connectivity characteristics). We believe that by introducing more parameters we would have given the impression of a more realistic network but our results would have been of lesser value since we would have been able to fit divergent patterns solely by fine tuning within a large parameter space. Naturally, our model uses certain simplifying assumptions. The Laplacian operator implies that the dynamic system is conservative and that the diffusion is also perfectly symmetric. However the brain is not a symmetric system, there is a directionality of the flow. Furthermore, the leakage rate along each neuron or brain region is not perfectly balanced with the flow it receives. Future studies will need to relax and test the assumptions imposed by the model.

### Graph diffusion and small worldness

For a wide range of species from C. elegans to humans, neuronal connections have been shown to form small world networks, with small path length and high clustering coefficient^2,37,49^. Adaptive rewiring based on diffusion when driven by the network’s own functional connectivity, leads to similar small world structure. Networks evolve to small worlds for a wide range of rewiring rates and random rewiring probabilities (Fig. 2). This is by and large because diffusion shapes the initial random network into a more structured connectivity pattern with high clustering coefficient while at the same time it maintains high efficiency-or else small path length (Fig. S2).

Adaptive rewiring based on diffusion is an abstraction of the consequences of Hebbian learning, where connections with heavy traffic are strengthened whereas the ones with less activity are weakened or pruned. Hebbian processes may lead to changes in the connectivity pattern of higher cognitive regions throughout childhood and adolescence. For example, using resting state functional connectivity MRI on children and adults, Fair and colleagues showed that with age, short-range functional connections between regions that are involved in top-down control regress whereas some long-range functional connections develop or increase in strength^50^. This process leads the connectome of the regions involved mostly in higher cognitive functions to have a small world structure^51^.

### Rewiring rate and network topology

Even though small-worldness is a key property of biological systems, these systems may have diverging topologies because of differentially weighted trade-offs between multiple constraints^52^. Diffusion-based models show topological variety across the range of the rewiring rate parameter τ. Across a wide range of rewiring rates, adaptive rewiring leads to approximately the same small world values, but different topological structures (Fig. 3). For smaller τ values (faster rewiring rate) the emerging network is modular (Fig. 4), with dense connectivity within clusters but sparse between them. For larger τ values (slower rewiring rate), we obtain centralized topologies where a subset of nodes are acting as hubs (Fig. 5C). Both of these patterns are present in the brain. Clustered neuronal modules facilitate local computations, an example being cortical minicolumns, the highly structured functional patterns in the sensory cortex. Neurons within a minicolumn and typically across the layers are densely connected, but tangential ones, across the layers to other minicolumns are sparsely connected^53^. On the other hand, centralized connectivity patterns resemble brain modules that receive and integrate a distributed set of neural inputs, for example the different association areas in the cortex^7,54^.

In the centralized regime, all types of networks show a similar degree distribution that is approximated by a power law distribution with an exponent of 1.7, for both binary and weighted networks (Fig. S3). Diffusion tractography^55^ and fMRI methods^56^ have shown a degree distribution between cortical and subcortical regions that follow an exponentially truncated power law with exponents of 1.66 and 1.80 respectively. Brain functional connectivity inferred from fMRI in different tasks shows a scale free distribution with a power law exponent of 2^57^. Given the simplicity of our model, we consider it to provide a surprisingly good approximation to the empirically observed degree distributions.

### Rich club structure

Brain networks at different levels and for different species have been shown to be topologically rich club^25,58,59^, that is, regions or neurons acting as hubs are also preferentially connected among themselves. Rich club connections in the brain constitute its backbone, communicating information from diverse functional modules. Empirical evidence has indicated that rich club connections extend over long distances forming a high capacity structure that facilitates global communication^25^.

We found that the nodes of both the normal and lognormal networks show topological rich club behavior for fast rewiring rates, but also for rewiring rates in the transition range and to a much lesser degree for slow rewiring rates (Fig. 6). Their corresponding weighted rich club index, which measures to what extent larger weights are attached to the connections within the rich club, was either at or below baseline (Fig. 7). This interesting contrast between topological and weighted rich clubs is in line with physiological data on the human brain (Fig. S8)^24,25^.

## Conclusion

The brain is responding continuously to a changing environment by strengthening or adding new connections and weakening or pruning existing ones. We tested whether an abstract rewiring model acting on weighted networks can reproduce graph properties found in the brain. Indeed, the model adaptively rewires an initially randomly connected network into a more structured one, with properties akin to the human brain such as small worldness and rich club structure. The adaptive changes made to the network follow heat diffusion, an abstract representation of brain functional connectivity. Moreover, depending on a parameter of the model, the rewiring rate, either modular or centralized connectivity patterns emerge, both found across different regions of the brain. For a narrow range of intermediate rewiring rates, the transition range, networks develop a full range from modular to centralized connectivity patterns. Weighted networks following rewiring in the modular and transition range are topologically rich club for a range of degrees, however the larger weights do not preferentially cluster in the rich club network. This combination of results has been shown in physiological studies. Overall, we show that rewiring based on heat diffusion is a parsimonious model suitable for representing the plastic changes taking place in the differentially weighted connections of the human brain.

## Supplementary information

Supplementary Information.
