# Supplementary material for: Adaptive rewiring evolves brain-like structure in weighted networks
Source: Sci Rep. 2020 Apr 8;10:6075. doi: 10.1038/s41598-020-62204-7 (PMC7142112; doi:10.1038/s41598-020-62204-7)
Supplement: Supplementary file 1 — Supplementary Information. [file 41598_2020_62204_MOESM1_ESM.docx]

# Supplementary Information:

# Adaptive rewiring evolves brain-like structure in weighted networks

Ilias Rentzeperis^1*^, Cees van Leeuwen^1,2^

^1^KU Leuven, Belgium

^2^ University of Technology Kaiserslautern, Germany

^*^Corresponding author:

E-mail: ilias.rentzeperis@gmail.com

# S1 Appendix

The heat kernel, *h(τ)*, is an *n*X*n* matrix (with *n* being the number of nodes in the graph) that describes the flow of heat or of any diffusing agent across the graph edges. It can provide information about the path length distribution of the graph. For small *τ* values, the heat stored in each node is diffused to its immediate neighbors (neighbors with larger edge weights are affected more); for larger *τ* values, the heat spreads further (Fig. S1). The heat stored in the nodes is found by summing the rows or the columns of *h(τ)*.

#

**Figure S1. As τ increases diffusion spreads more globally in the network.**

The heat kernel, *h(τ)*, of a randomly connected weighted network (the weights having a normal distribution) for different *τ* values. As *τ* increases, diffusion from each node affects more distant nodes.

# S2 Appendix

Below is the formulation of the heat diffusion rewiring that reflects our code implementation that is publicly available (<https://github.com/rentzi/netRewireAnalyze>). The algorithmic implementation is essentially the same as the one in Jarman and colleagues^1^, extended for application to both binary and weighted networks.

Starting with a random Erdös–Rényi type with *|V| = n* nodes and $\left| E \right|=\left\lfloor2\frac{\log\left( n \right)}{n}n(n-1) \right\rfloor$ connections- the latter guaranteeing the random network is connected ^2^- the rewiring algorithm proceeds as follows:

Step 1: Select, with uniform random probability, a node, *k* from the set of nodes in the graph that are of nonzero degree but also not connected to all other nodes $\left( k\in V \right| 0<d_{k}<n-1)$.

Step 2: With probability p_random_, go to step 2.1 (random rewiring), otherwise (1- p_random_) go to step 2.2 (heat diffusion rewiring). In both cases, select node *j_1_* from the set of nodes that are not connected to *k*, $(j_{1}\in\left\{ j\in V | \left( j,k \right)\notin E \right\})$, and node *j_2_* from the set of nodes that are connected to *k*, $(j_{2}\in\left\{ j\in V | \left( j,k \right)\in E \right\})$. Delete the edge, (*k, j_2_*) and add the edge (*k, j_1_*). In the case of weighted networks use the weight of the previously connected edge (*k, j_2_*) for the edge (*k, j_1_*).

Step 2.1: For both *j_1_* and *j_2_* the selection is random and uniform among the elements of each set described above. We do not calculate the heat kernel, *h(τ)*. Skip step 2.2.

Step 2.2: Calculate the heat kernel, *h(τ)*, of the adjacency matrix. *j_1_* is selected such that from all the nodes not connected to *k*, it is the one with the highest heat transfer with *k*. *j_2_* is selected such that from all the nodes connected to *k*, it is the one with the lowest heat transfer with *k*. τ stays fixed throughout the rewiring. In mathematical terms, we can express the statements above as follows:

$$j_{1}={argmax}_{\left( k,j \right)\notin E, k\neq j}h_{kj}\left( \tau\right) (1)$$

$$j_{2}={argmin}_{\left( k,j \right)\in E, k\neq j} h_{kj}\left( \tau\right) (2)$$

Step 3. Go back to Step 1 until a preset number of rewiring iterations has been reached.

# S3 Appendix

The *clustering coefficient*, C, measures the local cohesiveness of a network by quantifying the extent to which two nodes both adjacent to a common node are themselves connected to each other. The *weighted clustering coefficient*, *C_w_*, includes the weight of the edges forming these triplets. It is defined as:

$$C_{w}= \sum_{i=1}^{n} c_{i}^{w} (3)$$

where $c_{i}^{w}$ measures the clustering coefficient for node *i* and is expressed as:

$$c_{i}^{w}=\frac{1}{s_{i}(d_{i}-1)}\sum_{j,h} \frac{w_{ij}+ w_{ih}}{2}\alpha_{ij}\alpha_{ih}\alpha_{jh} (4)$$

*s_i_* is the strength and *d_i_* the degree of node *i*, *w_ij_* the weight of the connection between nodes *i* and *j*, and *α_ij_* a binary index that is 1 if *w_ij_* >0, and 0 otherwise^3^. Equation 3 also applies to binary networks.

The path length, *L_ij_*, defined as the distance between node pairs *i* and *j* along their shortest paths, is a popular metric in network analysis. Instead of *L* we opted to use a related metric, efficiency (*E*), which measures how efficient is the information flow within the network. We believe *E* is a more fitting characterization in the context of the brain. Efficiency is defined as the inverse of path length:

$$E_{ij}=\frac{1}{L_{ij}} (5)$$

*E* has important ramifications for a network’s behavior. For example, in a computational unit such as a visual module, a large-valued *E* suggests that the output of local computations can be relayed fast to other components of the network, enabling global computations such as normalization. In the case of binary networks, all connections carry the same weight, so we can directly measure the minimum number of jumps from each pair of nodes and invert it. For weighted networks where weights represent connection strength, it is reasonable to assert that the larger the weight between two nodes the greater their efficiency, so that, for instance, if we double the weight of an edge we effectively double its efficiency. The average efficiency of a network is defined as:

$$E_{ave}= \sum_{i\neq j} E_{ij} (6)$$

A network is *small world* if nodes connected to a common node are likely to be connected as well (high *C*), but at the same time any node can reach any other node in a few steps (small *L*). Accordingly, the measure of small worldness of a network is typically defined as:

$$S= \frac{\frac{C}{C_{random}}}{\frac{L}{L_{random}}} (7)$$

where *C* and *L* are the average clustering coefficient and path length of the network, and *C_random_* and *L_random_* are the ones, respectively, for a random network with the same number of nodes and connections. As discussed above, small path length implies high efficiency. For our purposes we use the following definition of small worldness with is equivalent to equation 7:

$$S= \frac{C}{C_{random}} \frac{E}{E_{random}} (8)$$

A network is considered small world when *S*>1.

To obtain a measure of network *modularity*, we used the spectral algorithm for community detection introduced by Newman^4^, which identifies communities within a network and assigns to this community division a modularity index (*Q*). The index *Q* gives a measure of the density of the interconnections within and between the communities, compared to a randomly connected graph with the same degree distribution. Hence, the greater *Q* is, the denser the connections within- and the sparser between communities. To identify the communities, the algorithm initially divides a network into two clusters, by computing the eigenvector corresponding to the largest eigenvalue of the modularity matrix, *B*, and assigning the nodes to one or the other group by the sign of the elements in the eigenvector. The entries of *B* are defined as:

$$B_{ij}=A_{ij}-\frac{s_{i}s_{j}}{2m} (9)$$

where *s_i_* is the strength of node *i*, and *m* is the total strength of the network. For this division into two groups *Q* is then:

$$Q=\frac{1}{4m}g^{T}Bg (10)$$

where *g_i_* is 1 is node *i* belongs to group 1, and -1 if it belongs to group 2. The algorithm iteratively continues to subdivide the groups and add the additional contribution *ΔQ*. It stops when any additional split provides a negative *ΔQ*. For a complete description of the iterative algorithm see Newman^4^.

A network is *assortative* if nodes with similar characteristics are preferentially connected. In our study the characteristic is degree. The assortativity coefficient^5^ formally expresses this property as:

$$r= \frac{\sum_{ij} {(A}_{ij}- {s_{i}s_{j}}/{2m})d_{i}d_{j}}{\sum_{ij} {(s}_{i}\delta_{ij}-{s_{i}s_{j}}/{2m})d_{i}d_{j}} (11)$$

where *A_ij_* is the weight of the connection between the *i* and *j* nodes, *s_i_* is the strength of the *i*-th node, *m* is the total strength of the network and *d_i_* is the degree of node *i*. *r* can take values between 1 and -1, with 1 signifying a perfectly assortative network and -1 a perfectly disassortative one. The equation is applicable to both binary (where *s_i_* is equal to *d_i_*) and weighted network and is equivalent to the equations found in other studies for weighted networks^6,7^.

*Rich club*^8,9^ refers to high degree nodes preferentially connecting to each other. The topological rich club coefficient, *Φ(k)*, disregards connection weights. It quantifies the tendency of the subgroup of nodes with degree greater than *k* to connect with each other. It is expressed as the ratio of the existing connections between the nodes with degree greater than *k* over the maximum possible connections between them:

$$\Phi\left( k \right)= \frac{2M_{>k}}{N_{>k}(N_{>k} -1)} (12)$$

where *N_>k_* is the number of nodes with degree greater than *k*, and *M_>k_* is the number of connections between them. The rich club coefficient naturally increases with increasing *k*, even in randomly connected networks. We therefore normalize *Φ(k)*, with the coefficient, *Φ_random_(k)*, obtained from a random network with the same number of nodes, connections, and degree distribution as the original one. We construct the random network by reshuffling, i.e. by performing 100**M_>k_* double edge swaps from the original network. A double edge swap involves randomly choosing two connections a-b and c-d, and replacing them with the connections a-c and b-d. If the later connections already exist, then the algorithm does not count that iteration and chooses again two random connections^10^. The normalized rich club coefficient is expressed as:

$$\Phi_{norm}=\frac{\Phi(k)}{\Phi_{random}(k)} (13)$$

For the weighted rich club coefficient, *Φ_w_(k)*, the node selection is based, similarly to *Φ(k)*, on the degree k. *Φ_w_(k)* is expressed as:

$$\Phi_{w}\left( k \right)= \frac{W_{>k}}{\sum_{i=1}^{E_{>k}} w_{i}^{ranked}} (14)$$

where *W_>k_* denotes the sum of the connection weights between the nodes with degree greater than *k*, *E_>k_* is the number of connections between them, and *w^ranked^* , the weights in the network in descending order. To normalize the weighted rich club coefficient, we use *Φ_W,random_(k)*, which has the same topology as the original network but reshuffled edge weights^11,12^. The weighted normalized rich club is then:

$$\Phi_{W,norm}=\frac{\Phi_{w}(k)}{\Phi_{W,random}(k)} (15)$$

# S4 Appendix

We performed pairwise correlations (Pearson coefficient; ρ) along the τ dimension, separately for each of the 100 iterations and p_random_ bins (demarcated by pairs of p_random_ values 0.1 apart), in order to quantify the dependencies between efficiency (*E*), clustering coefficient (*C*) and small-world (*S*) metrics. Qualitatively, *C* shows a similar pattern to *S*: steepest increase with respect to τ for binary networks, and further increasingly moderately for both the networks with normal and lognormal weight distributions (Fig. S2A). *C* is perfectly correlated with *S* (average across iterations and probability bins was ρ_binary_ = 0.91, ρ_normal_ = 0.96, ρ_lognormal_ = 0.97).

By contrast, *E* is not a monotonically decreasing function of τ but reaches a minimum and subsequently reverts back to values close to those of random connectivity networks; the minimum is reduced to an almost flat curve for p_rand_ >0.4 (Fig. S2B). Overall, across τ, *E* was negatively correlated with *C*, for all networks (ρ_binary_ = -0.42, ρ_normal_ = -0.52, ρ_lognormal_ = -0.31). The correlations, along with those of the *S* values, indicate that diffusion-based rewiring increases small worldness by increasing clustering at a greater rate compared to the decrease in efficiency (increase in path length when considering the classical definition of small worldness, equation 7), something that is more accentuated for larger random rewiring probabilities (compare Fig. S2A with S2B).

**Figure S2. An increase in *C* as a function of τ is accompanied by a more moderate decrease in *E*.**

A. *C* as a function of τ for different p_random_ (0, 0.4, 0.8). B. Same as A for the *E* metric. The s.e.m. vertical lines are not visible because they do not exceed the markers.

# S5 Appendix

To probe the heavy-tailed degree distributions of the centralized networks, we used p_random_ = 0.2, and τ_binary_ = 5, τ_normal_ = 5, τ_lognormal_ = 7 as representative τ values for centralized networks. Using log-likelihood ratios, we determined whether they best fit a power-law, lognormal or exponential distribution. We first compared the lognormal with the power-law fit, and then the better fit of the two with that of the exponential distribution. We repeated this process for 100 different realizations of the network. In over 90% of the cases either power-law or lognormal were a better fit compared to exponential distributions, with no significant difference between lognormal and power-law. The fits were adjusted so that they optimally accommodate the tail of the distribution, i.e. bins with the smaller degrees were not included, if they did not improve the fit. For the optimal range of degrees at the tail end of the distributions, the lognormal and power-law fits were almost identical (Fig. S3, fits on the left side of the plots). We performed the same analysis for networks of 1000 nodes with the same density of edges as with the 100 nodes (this time for one iteration due to the computational load) to probe whether the fit changes when the maximum allowable connections of a node increases by an order of magnitude with similar conclusions (but worse fits). Power law distribution functions are of the form *P(k) ~ k^-α^*. For all networks the exponent α was the same up to a decimal (*α* = 1.7). The strength distributions of the normal and lognormal networks again showed a similar behavior to their degree counterparts with the only difference being that the distribution decayed faster (exponent *α* varied between 2 and 2.6).

**Figure S3. Power law and lognormal fits are better fits of the degree distribution of centralized networks compared to the exponential fit.**

Log-log plot of the degree distribution of an example 100 node network and a 1000 node network along with their power-law and lognormal fits for (A) binary, (B) normal and (C) lognormal network

**Figure S4. Modularity profiles are the same with Fig. 4A when calculated from a multilevel algorithm**^13^**.**

A. Modularity index *Q* calculated from a multilevel algorithm for networks with normal weight distributions for different values of the system parameters, τ and p_random_. B. The same for networks with lognormal weight distributions.

**Figure S5. Networks are in two assortative states: a low and a disassortative one.**

The assortativity coefficient as a function of τ for binary, normal and lognormal networks. p_random_ = 0.2. The s.e.m. lines do not exceed the markers.

**Figure S6. The number of nodes in a rich club as a function of degree threshold decreases at different rates for separate τ ranges, with the modular range showing the steepest decrease and the centralized the most gradual one.**

A. Number of nodes with degree greater than *k* for modular networks (τ_binary_ = 2, τ_normal_ = 3, τ_lognormal_ = 4.5). The values show, for a given degree threshold, how many nodes constitute the rich club subnetwork. The black horizontal line is at 3 (nodes with degree greater than *k*). B. Same as A but for networks in the transition range (τ_binary_ = 4.1, τ_normal_ = 4.15, τ_lognormal_ = 5.5). C. Same as A and B but for centralized networks (τ_binary_ = 5, τ_normal_ = 5, τ_lognormal_ = 7). In all cases p_random_ = 0.2. The s.e.m. lines do not exceed the markers.

**Figure S7. Φ(k) deviates significantly from Φ_random_(k) for all τ ranges albeit with different divergence profiles.**

A. Φ(k), Φ_random_(k) and Φ_norm_(k) as a function of degree threshold (k) for binary (left), normal (center) and lognormal (right) modular networks (τ_binary_ = 2, τ_normal_ = 3, τ_lognormal_ = 4.5). The green markers above the curves indicate the *k* values for which Φ(k) is significantly greater than Φ_random_(k). We estimated significance with a permutation test: for a given *k*, we measured the ground truth that is the difference between the means (from all the iterations) of Φ(k) and Φ_random_(k). We then randomly shuffled the data split them and compute the difference in their mean. We repeated this process 10000 times and collected a distribution of differences. The proportion of permuted differences that were greater than our ground truth was our *p* value. We considered a difference significant if *p* < 0.001. B. Same as A but for networks in the transition range (τ_binary_ = 4.1, τ_normal_ = 4.15, τ_lognormal_ = 5.5). C. Same as A and B but for centralized networks (τ_binary_

= 5, τ_normal_ = 5, τ_lognormal_ = 7). In all cases p_random_ = 0.2. Vertical lines indicate s.e.m.

**Figure S8. The connectivity pattern of the white tracts in the human brain is topologically rich club; its weighted counterpart, however, is not**^taken from 11^**.**

A. The topological rich club of the white tracts in the human brain as measured with diffusion tensor imaging data (40 subjects). B. The corresponding weighted rich club.

# References

1. Jarman, N., Steur, E., Trengove, C., Tyukin, I. Y. & van Leeuwen, C. Self-organisation of small-world networks by adaptive rewiring in response to graph diffusion. *Sci. Rep.* **7**, (2017).

2. Erd\Hos, P. & Rényi, A. On the evolution of random graphs. *Publ Math Inst Hung Acad Sci* **5**, 17–60 (1960).

3. Barrat, A., Barthelemy, M., Pastor-Satorras, R. & Vespignani, A. The architecture of complex weighted networks. *Proc. Natl. Acad. Sci.* **101**, 3747–3752 (2004).

4. Newman, M. E. J. Modularity and community structure in networks. *Proc. Natl. Acad. Sci.* **103**, 8577–8582 (2006).

5. Newman, M. E. J. Mixing patterns in networks. *Phys. Rev. E* **67**, (2003).

6. Leung, C. C. & Chau, H. F. Weighted assortative and disassortative networks model. *Phys. Stat. Mech. Its Appl.* **378**, 591–602 (2007).

7. Farine, D. R. Measuring phenotypic assortment in animal social networks: weighted associations are more robust than binary edges. *Anim. Behav.* **89**, 141–153 (2014).

8. Colizza, V., Flammini, A., Serrano, M. A. & Vespignani, A. Detecting rich-club ordering in complex networks. *Nat. Phys.* **2**, 110 (2006).

9. Zhou, S. & Mondragon, R. J. The Rich-Club Phenomenon in the Internet Topology. *IEEE Commun. Lett.* **8**, 180–182 (2004).

10. Towlson, E. K., Vertes, P. E., Ahnert, S. E., Schafer, W. R. & Bullmore, E. T. The Rich Club of the C. elegans Neuronal Connectome. *J. Neurosci.* **33**, 6380–6387 (2013).

11. Alstott, J., Panzarasa, P., Rubinov, M., Bullmore, E. T. & Vértes, P. E. A unifying framework for measuring weighted rich clubs. *Sci. Rep.* **4**, 7258 (2014).

12. Serrano, M. Á. Rich-club vs rich-multipolarization phenomena in weighted networks. *Phys. Rev. E* **78**, (2008).

13. Blondel, V. D., Guillaume, J.-L., Lambiotte, R. & Lefebvre, E. Fast unfolding of communities in large networks. *J. Stat. Mech. Theory Exp.* **2008**, P10008 (2008).
